# Supplementary material for: “Moving from one environment to another, it doesn’t automatically change everything”. Exploring the transnational experience of Asian-born gay and bisexual men who have sex with men newly arrived in Australia
Source: PLoS One. 2020 Nov 20;15(11):e0242788. doi: 10.1371/journal.pone.0242788 (PMC7678978; doi:10.1371/journal.pone.0242788)
Supplement: Attachment — Submitted filename: Response to Reviewers_Final.docx [file pone.0242788.s002.docx]

# Editorial Requests and Comments

Thank you for submitting your manuscript to PLOS ONE. You have made considerable revisions in response to the reviewers' and editor's comments, and the manuscript is much improved. After careful consideration, we feel that it has merit but does not fully meet PLOS ONE’s publication criteria as it currently stands. For one, PLOS ONE does not copy edit manuscripts, so the following corrections need to be made. Additionally, a few statements need to be revised. Therefore, we invite you to submit a revised version of the manuscript that addresses the points raised during the review process (line numbers refer to revised version with track changes):

1. Line 221: Should be 2e. it jumps from 2d. to 2f.

**Response:** Thank you, this has been changed

2. Line 916: Should be: Skills 2e.

**Response**: Thank you, this has been changed

3. 1080: You refer to a “separate paper” with no reference. At least you should reference this as an “unpublished manuscript”. However, given it has not completed peer-review, you should also cite published articles that address social support among GBMSM, of which there are many:

See for ex. an earlier systematic review that includes MSM in the context of HIV risk, & others:

Qiao S, Li X, Stanton B. Social support and HIV-related risk behaviors: a systematic review of the global literature. *AIDS Behav*. 2014;18(2):419-441. doi:10.1007/s10461-013-0561-6

Saleh LD, van den Berg JJ, Chambers CS, Operario D. Social support, psychological vulnerability, and HIV risk among African American men who have sex with men. *Psychol Health*. 2016;31(5):549-564. doi:10.1080/08870446.2015.1120301

**Response**: Thank you for this advice. Where our ‘separate’ publication is mentioned we add that the manuscript is pending publication. For example,

Line 109: “The overall aim of this study was to explore HIV knowledge and prevention strategies used and preferred among newly-arrived Asian-born gbMSM, the results of which are pending publication in a separate paper”

Line 248: “Findings related to men’s HIV and STI knowledge and HIV prevention strategies are reported in a separate paper (as yet unpublished).”

Line 1117: “In our separate paper from this study (as yet unpublished), we discuss our findings that social support was a facilitator to sexual health within this group…”

Additionaly, in line with your comment about including published references for this citation we have amended the text, adding the suggested references:

“In our separate paper from this study (unpublished manuscript), we discuss our findings that social support was a facilitator to sexual health within this group, notably some participants’ positive experiences with local LGBTQI organisations that offer peer-led social support workshops. Previous research has also found that social support can impact sexual behaviours (1, 2). Increasing awareness of and opportunities to engage with peer-led services may be beneficial for men in this group in terms of bolstering their social support in Australia.”

4. 1082: fix the apostrophe. Change to participants’

**Response**: Changed

5. 1157: fix the apostrophe. Change to country’s

**Response**: Changed

6. 1161: This is qualitative research. You didn’t “measure” anything, so this statement sounds strange. In another place in the manuscript you similarly note that you did not "measure resilience". These statements should be revised as quantitaive assessment is not generally part of qualitative research; it is not specific to your study.

**Response**: Thank you for your comment. We have amended the text as below:

Line 1147: “While measuring resilience (i.e. the ability to recover after experiencing adversity) among our participants was outside the scope of this study, peer support is a known protective process that leads to the development of resilience”

And

Line 1232: “This study did not ask participants to describe the degree to which they identified with a particular ethnicity, but recent research has questioned the role of ethnic identity as a protective buffer for the stress of discrimination (3). Future research could investigate whether men in this group with stronger ethnic identity have ameliorated stress levels in the face of discrimination.“

7. 1220-1222: You cite one source that apparently supports the claim that the use of more than one coder is “generally prohibitive” due to “cost and effort”: “While ideal, this is generally prohibitive in qualitative research due to cost and effort, when compared with the standard convention of cross-checking by a researcher in a supervisory role [44].”

This statement is misleading and overstated as written. The use of one coder is not the norm for qualitative analysis (and there are many references available to this end; for ex., see below). Indeed this is why the specific item is included on the COREQ checklist (see No. 24). Generally two to three independent coders are recommended. You need to revise this statement to correctly indicate this, although *you* may have used only one coder due to concerns about “cost and effort” and that shortcoming may be characteristic of some qualitative research (“particularly in early-career contexts” [Campbell et al., 2013]). Another source indicates the importance of carrying reflexivity through the data analytic stage, which may help to mitigate (though not erase) bias.

See for ex.:

Campbell, J. L., Quincy, C., Osserman, J., Pedersen, O. K. (2013). Coding in-depth semistructured interviews: Problems of unitization and intercoder reliability and agreement. Sociological Methods & Research, 42, 294–320. [https://doi.org/10.1177/0049124113500475](https://webmail.mshc.org.au/owa/redir.aspx?C=9500cc67cf4a4e82babe498e30dc85ac&URL=https%3a%2f%2fdoi.org%2f10.1177%2f0049124113500475)

Church, Sarah, Michael Dunn, and Linda Prokopy. 2019. "Benefits to Qualitative Data Quality with Multiple Coders: Two Case Studies in Multi-coder Data Analysis." Journal of Rural Social Sciences, 34(1): Article 2. Available At: [https://egrove.olemiss.edu/jrss/vol34/iss1/2](https://webmail.mshc.org.au/owa/redir.aspx?C=9500cc67cf4a4e82babe498e30dc85ac&URL=https%3a%2f%2fegrove.olemiss.edu%2fjrss%2fvol34%2fiss1%2f2)

Lacy, Stephen; Watson, Brendan R.; Riffe, Daniel; and Lovejoy, Jennette, "Issues and Best Practices in Content Analysis" (2015). Communication Studies Faculty Publications and Presentations. 8. [http://pilotscholars.up.edu/cst_facpubs/8](https://webmail.mshc.org.au/owa/redir.aspx?C=9500cc67cf4a4e82babe498e30dc85ac&URL=http%3a%2f%2fpilotscholars.up.edu%2fcst_facpubs%2f8)

Tufford L, Newman P. Bracketing in Qualitative Research. *Qualitative Social Work*. 2012;11(1):80-96. doi:[10.1177/1473325010368316](https://webmail.mshc.org.au/owa/redir.aspx?C=9500cc67cf4a4e82babe498e30dc85ac&URL=https%3a%2f%2fdoi.org%2f10.1177%2f1473325010368316)

[e.g., Walther et al. (2013) suggested IRR as a means to “mitigate interpretative bias” and ensure a “continuous dialogue between researchers to maintain consistency of the coding” (p. 650). Miles and Huberman (1994) suggest that an IRR of 80% agreement between coders on 95% of the codes is sufficient agreement among multiple coders(Miles & Huberman, 1994)]

**Reponse**: Thanks for this insight. The reference that we cited in our manuscript in the line you are referring to (Barbour, 2001: <https://www.ncbi.nlm.nih.gov/pubmed/11337448>) does indeed caution against multiple people coding the entirety of the data, here is the excerpt from the text: “While I would caution against multiple coding of entire datasets (on the grounds of economy in both cost and effort), some element of multiple coding can be a valuable strategy. It can be useful to have another person cast an eye over segments of data or emergent coding frameworks, and this is a core activity of supervision sessions and research team meetings.”

We have now changed the text to remove the suggestion that multiple coders are not the norm:

Additionally, had two to three researchers coded the entirety of the data (cross-coding/multiple coding) instead of one researcher doing the majority of the analysis, this would have provided more rigor to the analysis. While it would have been ideal to undertake cross coding of the data using multiple coders, which would have further assisted in mitigating bias, this was not possible due to time and resource limitations. This approach serves as a valuable strategy where these limitations apply (4).

1.	Qiao S, Li X, Stanton B. Social support and HIV-related risk behaviors: a systematic review of the global literature. AIDS Behav. 2014;18(2):419-41.

2.	Saleh LD, van den Berg JJ, Chambers CS, Operario D. Social support, psychological vulnerability, and HIV risk among African American men who have sex with men. Psychol Health. 2016;31(5):549-64.

3.	Mossakowski KN, Wongkaren T, Hill TD, Johnson R. Does ethnic identity buffer or intensify the stress of discrimination among the foreign born and U.S. born? Evidence from the Miami-Dade Health Survey. J Community Psychol. 2019;47(3):445-61.

4.	Barbour RS. Checklists for improving rigour in qualitative research: a case of the tail wagging the dog? BMJ. 2001;322(7294):1115-7.
